# Differences in Ribosome Binding and Sarcin/Ricin Loop Depurination by Shiga and Ricin Holotoxins

**DOI:** 10.3390/toxins9040133

**Published:** 2017-04-11

**Authors:** Xiao-Ping Li, Nilgun E. Tumer

**Affiliations:** Department of Plant Biology, Rutgers, The State University of New Jersey, New Brunswick, NJ 08901, USA; tumer@aesop.rutgers.edu

**Keywords:** Shiga toxin 1, Shiga toxin 2, Stx1, Stx2, ricin, ribosome binding, depurination activity

## Abstract

Both ricin and Shiga holotoxins display no ribosomal activity in their native forms and need to be activated to inhibit translation in a cell-free translation inhibition assay. This is because the ribosome binding site of the ricin A chain (RTA) is blocked by the B subunit in ricin holotoxin. However, it is not clear why Shiga toxin 1 (Stx1) or Shiga toxin 2 (Stx2) holotoxin is not active in a cell-free system. Here, we compare the ribosome binding and depurination activity of Stx1 and Stx2 holotoxins with the A1 subunits of Stx1 and Stx2 using either the ribosome or a 10-mer RNA mimic of the sarcin/ricin loop as substrates. Our results demonstrate that the active sites of Stx1 and Stx2 holotoxins are blocked by the A2 chain and the B subunit, while the ribosome binding sites are exposed to the solvent. Unlike ricin, which is enzymatically active, but cannot interact with the ribosome, Stx1 and Stx2 holotoxins are enzymatically inactive but can interact with the ribosome.

## 1. Introduction

Shiga-toxin producing *E. coli* (STEC) infections can cause life threatening complications such as hemolytic uremic syndrome (HUS) or hemorrhagic colitis (HC), and are the leading cause of death from foodborne bacterial infections in children [1]. Shiga toxins (Stxs) are the primary virulence factors of STEC and belong to a group of proteins called type II ribosome inactivating proteins (RIPs). There are two main Stx types, designated Shiga toxin 1 (Stx1) and Shiga toxin 2 (Stx2), and within each are many subtypes. Stx1 and Stx2 contain a catalytically active A subunit and five copies of cell binding B subunits [2,3]. Stx1 and Stx2 share 55% and 57% amino sequence identity in the A and B subunits, respectively, and have similar molecular structures. The A subunits remove a universally conserved adenine from the sarcin/ricin loop (SRL) of the large ribosomal RNA and inhibit translation. The B subunits bind to a globotriaosylceramide (Gb3 or CD77) receptor on the cell surface and facilitate endocytosis. Ricin produced by the castor bean is another type II RIP. The A subunit of ricin (RTA) has similar enzymatic function and molecular structure to the A subunits of Stxs, while the B subunit of ricin (RTB) is functionally similar but structurally different and binds to different cellular receptors [2]. Residues important for the enzymatic activity of the A subunits are conserved among all RIPs [4]. Although Stx and ricin holotoxins are very toxic to eukaryotic cells, they are not active towards the ribosome and are activated after separation of the B subunits from the A subunits [5,6].

**Figure 1:**
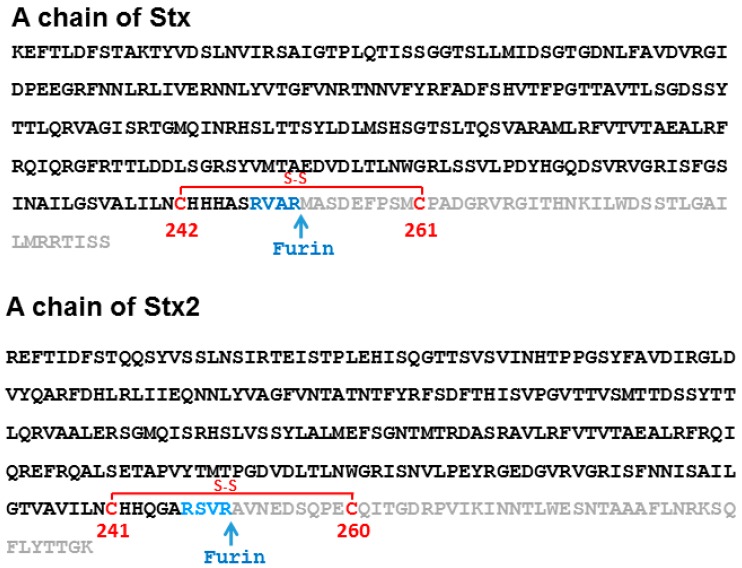
The sequences of mature A chains of *Shigella* Stx and Stx2. Furin recognition sites are shown in blue. The sequence before the furin cleavage site corresponds to the A1 subunits and the sequence after the furin cleavage site corresponds to the A2 subunits.

Stx1 and Stx2 are AB_5_ toxins consisting of one A subunit and a pentamer of B subunits. In *E. coli*, the genes encoding Stx1 or Stx2 A (A1 + A2) and B subunits are linked, but they are encoded by separate ORFs [7]. In ricin, the RTA and RTB are encoded by a single gene and synthesized as preproricin with a signal peptide preceding the A chain and a linker peptide between RTA and RTB [8]. The signal sequence and the linker peptide are cleaved during the maturation of ricin, but RTA and RTB remain linked together by a disulfide bond [9,10]. Stx1, Stx2 or ricin traffic from the endosome to the trans-Golgi network in a retrograde manner and reach the endoplasmic reticulum (ER) [5,6]. During this process, A1 and A2 chains of Stxs are cleaved by furin between R251 and M252 in Stx1 and between R250 and A251 in Stx2 (Figure 1) [11]. The furin cleavage sites are located in protease-sensitive loops. After furin cleavage, the A1 subunit and A2 chain remain connected by a disulfide bond and the B subunit remains connected to the A1 subunit through the A2 chain. In the ER, the disulfide bond is reduced by protein disulfide isomerase, releasing the A1 subunit of Stxs from the B subunit, and RTA from RTB. The A1 subunits of Stxs and RTA unfold and retrotranslocate out of the ER and refold into an active form in the cytosol [5,6].

Stx2-producing *E. coli* are more often associated with HUS than Stx1-producing *E. coli* but the reason for this is unclear [12,13]. We showed that the A1 subunit of Stx2 (Stx2A1) bound ribosomes more tightly, had higher activity and was more toxic than the A1 subunit of Stx1 (Stx1A1) [14]. RTA, Stx1A1 and Stx2A1 interact with the C-terminus of the ribosomal P-protein stalk to access the SRL [15,16,17,18,19]. The eukaryotic ribosomal P-protein stalk is a pentameric complex located on the 60S subunit in close proximity to the SRL. It consists of two P1/P2 dimers, which bind to uL10 (previously P0) to form uL10-(P1/P2)_2_ structure. The C-terminal sequence of the five P-proteins is exactly the same and is conserved among all eukaryotes. The ribosomal P-protein stalk together with the sarcin/ricin loop (SRL), are part of the GTPase associated center, which is responsible for the recruitment of translational GTPases and stimulation of factor-dependent GTP hydrolysis [20,21,22,23]. 

**Figure 2:**
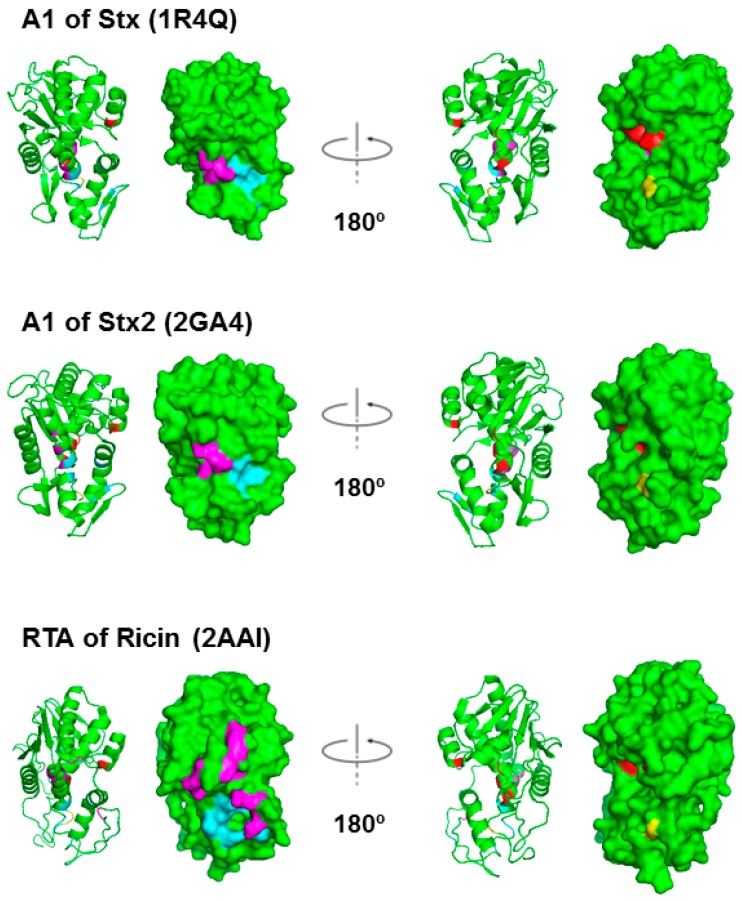
Structures of the StxA1, Stx2A1 and RTA shown as ribbon and as surface. The residues at the active site, Tyr76/Tyr77, Tyr114, Glu167 and Arg170 in Stx; Tyr80, Tyr123, Glu177 and Arg180 in RTA, are shown in red. The arginines that are critical for P protein interaction, Arg172, Arg176 and Arg179 in Stxs and Arg189, Arg191, Arg193, Arg196, Arg197, Arg234 and Arg235 in RTA, are shown in magenta. The residues that form a hydrophobic pocket and interact with the last six residues of the P-proteins, Gln173, Leu198, Ile224/Ile223, Phe226/Phe225, Leu233/Leu232, Ser235/Thr234 in Stx/Stx2 and Tyr183, Leu207, Phe240, Ile247, Pro250 and Ile251 in RTA, are shown in cyan [29]. Cys242/241 in Stx/Stx2 A1, which form a disulfide bond with Cys261/260 in Stx/Stx2 A2, and Cys259 in RTA, which forms a disulfide bond with Cys2 in RTB, are shown in yellow. Stx PDB ID: 1R4Q, Stx2 PDB ID: 2RA4 and ricin PDB ID: 2AA1. The structures were generated using PyMOL.

The X-ray crystal structures of Shiga toxin from *Shigella* (Stx), which differs only in one residue from *E. coli* Stx1, and *E. coli* Stx2 are resolved [24,25,26,27,28], but not the structures of the A1 subunits alone. Figure 2 compares the structure of RTA with the structures of StxA1 and Stx2A1 derived from their holotoxin structure. On the left, the residues in Stx and in RTA, which interact with the C-termini of ribosomal stalk P-proteins, are shown in magenta and in light blue. Rotating 180° along the y-axis, on the right, the active sites are shown in red. Cys242/241 in Stx/Stx2 A1, which forms a disulfide bond with Cys261/260 in Stx/Stx2 A2, and Cys259 in RTA, which forms a disulfide bond with Cys2 in RTB, are shown in yellow.

We showed that the RTA-ribosomal stalk interaction site is located on the opposite side of the active site [19]. In ricin holotoxin, the ribosome binding site of RTA was blocked by RTB [19]. Consequently, ricin holotoxin did not bind or depurinate the ribosome. However, the active site of ricin was not blocked by RTB and ricin holotoxin could depurinate the naked RNA [19].

The X-ray crystal structures of a peptide mimic of the conserved C-terminus of ribosomal P-proteins in complex with trichosanthin, a type I RIP, and RTA have been resolved [29,30,31]. The last six amino acids (P6) of the P-protein peptide bind to a hydrophobic pocket on RTA, which is blocked by RTB in the holotoxin. Several ribosome binding residues on the A1 subunits of Stxs have been identified [32]. As shown in Figure 2, the ribosome binding sites of Stxs are also located on the opposite face of the active site. Stx1 holotoxin displays almost no ribosomal activity in its native form. Why Stx1 and Stx2 holotoxins are not active on ribosomes remains unknown. Here we compare ribosome binding and depurination activity of Stx1 and Stx2 holotoxins with the A1 subunits of Stx1 and Stx2 using intact yeast ribosomes or a 10-mer RNA mimic of the SRL. We demonstrate that the A2 chain and the B subunit block the active site of Stx1 and Stx2, but not their ribosome binding site. Unlike ricin holotoxin, the ribosome binding sites of Stx1 and Stx2 holotoxins are accessible to the ribosome. These differences have implications for the development of antidotes against STEC.

## 2. Results

### 2.1. Stx1 and Stx2 Holotoxins Can Bind the Ribosome

**Figure 3:**
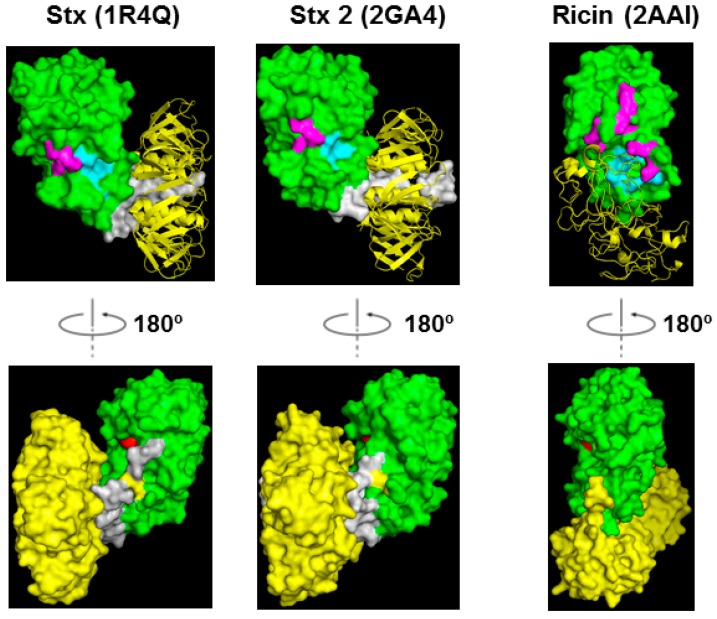
Structures of Stx, Stx2 and ricin holotoxin shown in ribbon and surface. The A1 subunits are shown in green, the A2 chains are shown in gray and the B subunits are shown in yellow. The labeling of the residues is the same as in Figure 1.

Computer modeling of Stx1A1 in complex with a peptide that mimics the last 11 amino acids of the ribosomal P-stalk proteins (P11) suggested that the ribosomal stalk binding site and the active site are located on opposite sides of the protein [30]. Our recent study showed that point mutations at Arg172 or Arg176 located on the opposite face of the active site (Figure 1 and Figure 3) disrupted the ribosome and ribosomal stalk interaction of Stx1A1 and Stx2A1, but did not affect their depurination activity on RNA, indicating that their active sites were intact and were located on the opposite face of the ribosome binding site [32]. Figure 3 shows the structure of Stx holotoxins in comparison with ricin. In Stxs, five B-subunits form a doughnut shape and A2 chains insert into the doughnut hole. The position of the B subunits is different between Stxs and ricin. The pentameric B subunits are docked on the active site of StxA1 and Stx2A1 and block the active site, while the ribosome binding site is exposed in Stx and Stx2 holotoxins. However, in ricin holotoxin the RTB covers the ribosome binding site of RTA while the active site of RTA is exposed.

**Figure 4:**
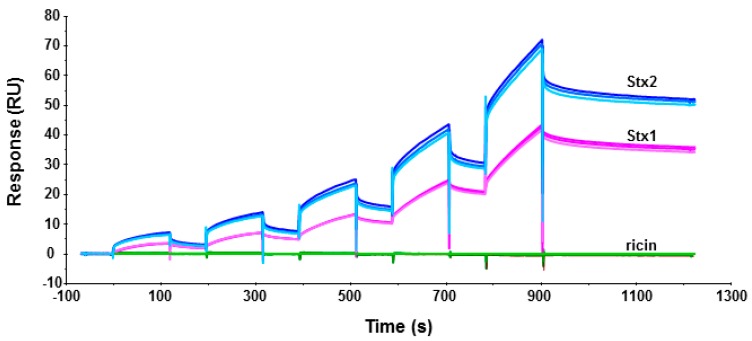
Interaction of yeast ribosomes with Stx1 (magenta), Stx2 (blue) and ricin (green) holotoxins. Toxins were immobilized on a CM5 chip. Ricin was immobilized on Fc2 at 1873 RU, Stx1 was immobilized on Fc3 at 2541 RU, and Stx2 was immobilized on Fc4 at 1853 RU. Fc1 was activated and blocked as a control. Ribosomes were passed over the surfaces at 2.5, 5, 10, 20 and 40 nM. The running buffer contained 10 mM Hepes, pH 7.4, 150 mM NaCl, 5 mM MgCl_2_, 50 µM EDTA and 0.003% Surfactant P20. The surface was regenerated with one-minute-injection of 3 M NaCl for three times. The interaction was measured at 25 °C. The signals were normalized to the ligand level of 1000 RU. The experiment was replicated three times.

To determine if Stx1 and Stx2 holotoxins can bind ribosomes, we immobilized Stx1, Stx2 and ricin on a CM5 chip of Biacore T200 and passed yeast ribosomes over the surface. As shown in Figure 4, both Stx1 and Stx2 could interact with yeast ribosomes. The binding levels of the interaction were ribosome concentration-dependent. In order to observe the interaction signal of Stx1, we immobilized 37% more Stx1 (2541 RU for Stx1 and 1853 RU for Stx2) on the chip. When the same concentration of ribosomes was passed over the surface, the binding level of Stx2 was still about 90% higher than Stx1 at 2.5 nM and 66% higher than Stx1 at 40 nM ribosome concentration. These data indicate that Stx1 and Stx2 holotoxins can bind ribosomes, and Stx2 holotoxin has higher affinity for the ribosome than Stx1 holotoxin. Similar results were observed when Stx1A1 and Stx2A1 interacted with the ribosome [14]. There was no detectable interaction between the ribosome and ricin holotoxin, as previously observed [19].

### 2.2. Stx Holotoxins Are Inactive Towards the Ribosome, but Are Active After Separation of the A1 from the A2 Chain and the B Subunit

**Figure 5:**
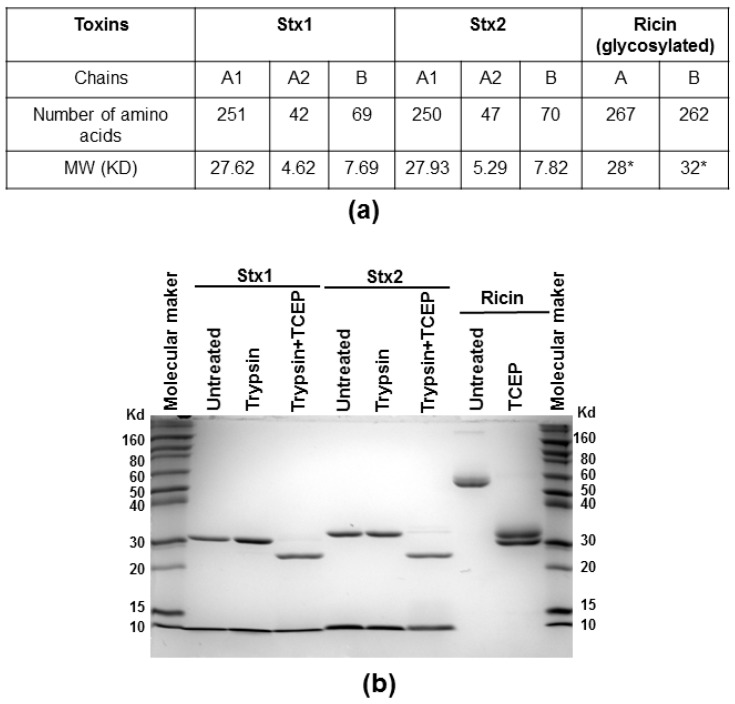
Molecular weight of the subunits of Stx1, Stx2 and ricin (**a**) and SDS-PAGE analysis (**b**); Stx1 and Stx2 (7 µg) were treated with trypsin with or without TCEP, and ricin (7 µg) was treated with TCEP at 25 °C for 2 h and separated on a 12% SDS-PAGE. The gel was stained with Coomassie blue. The protein marker is Invitrogen Novex® sharp pre-stained protein marker. * Molecular weight is affected by glycosylation in both A and B subunits of ricin.

We measured the depurination activities of Stx holotoxins before and after treatment with trypsin and TCEP (Tris (2-carboxyethyl) phosphine hydrochloride) to separate the A1 from the A2 chain and the B subunit. Stx1 and Stx2 were treated with TPCK (*N*-tosyl-l-phenylalanine chloromethyl ketone)-treated trypsin, which can cleave at the furin sites, with or without TCEP, to reduce the disulfide bond. Ricin was treated with TCEP only. After treatment, the toxins were separated on non-reducing SDS-PAGE. As shown in Figure 5, the A (A1 + A2) subunits migrated at 31 and 32 kDa for Stx1 and Stx2, respectively, and the B subunits migrated at about 8 kDa without TCEP treatment. After TCEP treatment, the A1 and A2 subunits were separated and the A1 subunits migrated at 28 kDa. The A2 subunits of 5 kDa were not observed on SDS-PAGE, possibly because their amounts were low. Ricin holotoxin migrated to about 60 kDa. The A and B subunits were separated after TCEP treatment with molecular weights around 30 and 32 kDa, respectively.

**Figure 6:**
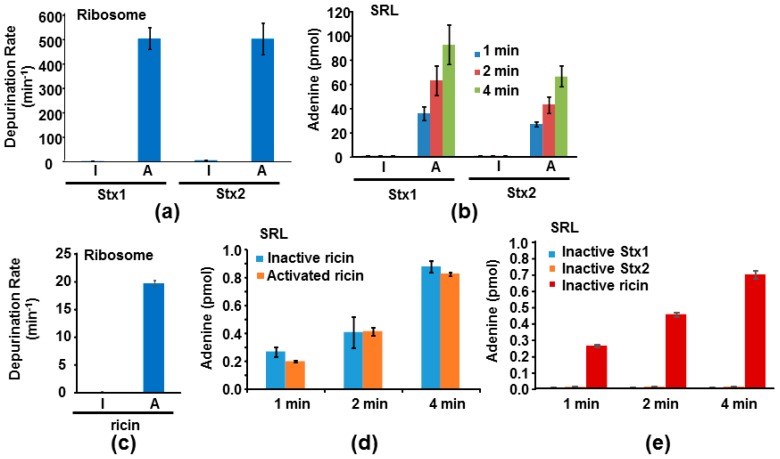
Depurination of yeast ribosomes (**a**,**c**) and SRL mimic RNA (**b**,**d**,**e**) by Stxs (**a**,**b**,**e**) and ricin (**c**,**d**,**e**). (**a**,**c**) Stx holotoxins (I for inactive) were activated by trypsin and TCEP (Tris (2-carboxyethyl) phosphine hydrochloride) treatment to release the A1 subunit from the A2 chain and the B subunit (A for active) and ricin holotoxin (I for inactive) was activated by TCEP treatment to release the A subunit from the B subunit (A for active). Ribosomes were used at 0.5 µM, Stxs were used at 0.2 nM and ricin was used at 1 nM. The reaction was incubated at 25 °C. The adenine released was measured by the enzyme coupled fluorescence assay for 15 min. The linear part of the reactions was used to calculate the activities. The depurination rate is shown as pmol of adenine released per pmol of toxin in one minute. (**b**,**d**) The SRL mimic RNA was used at 2 µM, Stxs were used at 5 nM and ricin was used at 40 nM. The reactions were incubated at 20 °C at pH 4.5. The depurination was stopped at 1, 2 and 4 min. The adenine released was measured by the enzyme coupled fluorescence assay. The amount of adenine released is shown as pmol of adenine released per pmol of toxins at the sampling time point. (**e**) The SRL mimic RNA concentration was 2 µM and the holotoxin concentrations were 1 µM. The reactions were incubated at 20 °C at pH 4.5. The depurination was stopped at 1, 2 and 4 min. The adenine released was measured by the enzyme coupled fluorescence assay. The amount of adenine released is shown as pmol of adenine released per pmol of toxins.

We previously measured the depurination kinetics of recombinant Stx1A1 and Stx2A1 from *E. coli* on ribosomes and on an SRL mimic RNA [14]. Both StxA1 and RTA can depurinate ribosomes at physiological pH but they depurinate RNA only at an acidic pH [14]. Using previously established conditions to measure depurination kinetics [14], we compared the depurination activities of Stx1 and Stx2 holotoxins with their activated forms where the catalytic A1 subunit was separated from the A2 chain and the B subunit. Stx1 and Stx2 holotoxins were not active towards the ribosome. However, after trypsin and TCEP treatment to release the A1 subunits, Stx1A1 and Stx2A1 were able to depurinate the yeast ribosome at 374-fold and 92-fold higher levels than Stx1 and Stx2 holotoxins, respectively, at 0.5 µM ribosome concentration (Figure 6a). A previous study examined the *k*_cat_ and *K*_m_ of Stx1 holotoxin on *Artemia salina* ribosomes before and after treatment with trypsin, urea and DTT [33]. Our results are consistent with this study in the requirement for activation, although this study used a different assay, different source of ribosomes, different method of activation, and much higher toxin concentrations [33].

We further examined the activity of Stx1 and Stx2 holotoxins with their activated forms toward a 10-mer RNA stem loop mimic of the SRL. Activated Stx holotoxins could depurinate the SRL mimic RNA, while the holotoxins were inactive (Figure 6b). These results are consistent with the structural data, indicating that the A2 chain and the B subunit block the active sites of Stx1 and Stx2 [24,25,26,27,28]. We have previously shown that ricin holotoxin could not depurinate the ribosome but can depurinate the naked RNA using a qRT-PCR assay to measure depurination [19]. Here, as a control, we measured the activities of ricin holotoxin toward the yeast ribosome and the SRL using a highly sensitive luminescent assay [34]. Consistent with the previously published data, ricin holotoxin could not depurinate yeast ribosomes unless it was reduced to separate the catalytic A subunit from the B subunit (Figure 6c). In contrast, both unreduced and reduced ricin holotoxin could depurinate the SRL mimic RNA with similar activity (Figure 6d). Stx1A1, Stx2A1 (pH 4.5) and ricin (pH 4.0) have different optimum catalytic pH on nucleic acid substrates [14,34,35]. We examined depurination of the SRL mimic RNA at the same pH (pH 4.5) in order to compare the activity of activated Stx1 and Stx2 holotoxins with ricin. At pH 4.5, A1 subunits of Stxs are about 100-fold more active than ricin holotoxin or RTA (Figure 6b,d). To determine if Stx1 and Stx2 holotoxins have any activity on the SRL mimic RNA at a very high concentration, we compared their activity with ricin holotoxin at 1 µM toxin concentration using the same conditions as in Figure 6b,d. As shown in Figure 6e, we could not detect any depurination activity of Stx1 and Stx2 holotoxins, even at this higher dose. In contrast, ricin holotoxin could depurinate the SRL at a similar level as that in Figure 6d. Ricin holotoxin could depurinate the SRL mimic RNA at 252-, 326-, and 364-fold higher levels than Stx1 and at 51-, 76-, and 114-fold higher levels than Stx2 at 1, 2 and 4 min, respectively (Figure 6e). 

In summary, the A2 chain and the B subunit block the active site in the Stx holotoxins, but leave the ribosome binding sites open. As a result, Stx holotoxins could not depurinate either the ribosome or the 10-mer RNA stem loop mimic of the SRL, even though they could interact with the ribosome. In contrast, in ricin holotoxin RTB covers the ribosome binding side of RTA and leaves the active site of RTA exposed. Ricin holotoxin could depurinate the SRL mimic RNA at low pH, but could not interact with the ribosome or depurinate the ribosome. These results explain why Stx1, Stx2 and ricin holotoxins are inactive towards ribosomes and show that the molecular basis for the inactivity of Stx1/Stx2 holotoxins differs from that of the ricin holotoxin.

## 3. Discussion

We previously showed that ricin holotoxin is not active on ribosomes because the ribosome binding site of RTA is blocked by the B subunit [19]. However, the molecular basis for the inactivity of Stx1 and Stx2 holotoxins towards ribosomes remained unknown. Here, we compared the ribosome binding and depurination activity of Stx1 and Stx2 holotoxins with the A1 subunits of Stx1 and Stx2 and demonstrated that the active sites of Stx1 and Stx2 are blocked by the A2 chain and the B subunit, while the ribosome binding sites are exposed. Although Stx1 and Stx2 holotoxins are enzymatically inactive, they are able to interact with the ribosome. Previous results indicated that the A1 subunit of Stx2 interacts with the ribosome with higher affinity than the A1 subunit of Stx1 [14]. Conserved 1 subunits for the rib arginines at the P-protein stalk binding site did not contribute to the differences in the affinity of the A osome [32]. We show here that Stx2 holotoxin also interacts with the ribosome with higher affinity than Stx1 holotoxin (Figure 4). Since the difference in ribosome binding previously observed for the A1 subunits [14] is also observed with the Stx1 and Stx2 holotoxins, the regions of the A1 subunits, which are covered by the A2 chain and the B subunit, are not likely responsible for the differences in the ribosome binding. These results suggest that residues at the active site of Stx2A1 and Stx1A1 do not contribute to the higher affinity of Stx2 for the ribosome. The surface charge and the distribution of the surface charge are different between Stx1A1 and Stx2A1 [32]. Our results suggest that surface charge differences in regions other that the active site or the stalk binding site contributes to the higher affinity of Stx2 for the ribosome. 

Although Stx2A1 is more active in depurination of the ribosome and the RNA than Stx1A1 [32], we did not observe this difference when the holotoxins were activated by trypsin and TCEP (Figure 6). As previously observed [14], the holotoxin is not a good choice for comparison of the depurination activities of Stx1 and Stx2. Stx1 and Stx2 holotoxins have to be activated by trypsin and TCEP treatment to separate the A2 chains and the B subunits from the A1 subunits. It is impossible to digest Stx1 and Stx2 equally with trypsin. Since trypsin cleaves the peptide chains at the carboxyl side of lysine and arginine, trypsin can cleave the toxins at other sites besides the furin cleavage site between A1 and A2, and inactivate them. Furthermore, during toxin purification, some of the toxin molecules are already cleaved between A1 and A2 subunits and the holotoxins can be activated to up to 80% of their maximal activity after treatment with only TCEP. Therefore, the activity measured after trypsin plus TCEP (or DTT) treatment does not necessarily reflect the total activity of the A1 subunits in the Stx holotoxins, but the activity of the A1 subunits remaining after the trypsin digestion. 

The X-ray crystal structure comparison of Stx2 holotoxin with *Shigella* Stx indicated some differences. While the active site of the A subunit was blocked by the A2 chain in the *Shigella* Stx, the active site of Stx2 holotoxin was accessible to a small substrate, adenosine [26]. However, it was predicted that while Stx2 holotoxin was active against adenosine, it was unlikely to bind to the SRL because the A2 chain and the B subunit would interfere with binding to the 28S rRNA [26]. Our results (Figure 6c,e) provide direct experimental evidence that the Stx1 and Stx2 holotoxins cannot depurinate the SRL or the ribosome because their active sites are blocked by the A2 chain and the B subunit. In contrast, ricin holotoxin could depurinate the SRL mimic RNA. However, depurination of the RNA occurs only at the low pH, but not at the physiologic pH. Ricin holotoxin could not interact with the ribosome or depurinate the ribosome due to the blockage of the ribosome binding site by RTB. These results demonstrate the molecular basis for the inactivity of ricin, Stx1 and Stx2 holotoxins towards ribosomes and suggest that deactivation of the toxins could be achieved by blocking the ribosome binding site as an alternative to the active site, since targeting the active site has not yielded an inhibitor with sufficiently high activity to date [36]. 

Although Stx1A1, Stx2A1 and RTA depurinate small RNA substrates at a low pH [14,34,35], at the physiologic pH, Stx1A1, Stx2A1 and RTA need to interact with the ribosomal P-proteins to catalyze depurination of the SRL. Stx1A1, Stx2A1 and RTA can depurinate the ribosome with high affinity. However, since the ribosome and the A subunits interact with each other electrostatically [32,37], the local concentration of the toxins around the ribosome can be high. In order to block ribosome interaction inhibitors that can bind either to the P-protein stalk interaction site or to the active site would require very high affinity. The molecular details of how Stx1A1 and Stx2A1 interact with ribosomes and the structures of the C-termini of P-proteins in complex with the toxins could lead to the identification of inhibitors that can disrupt ribosome interactions and thereby offer new opportunities for antidote development.

## 4. Materials and Methods 

### 4.1. Materials

Stx1 and Stx2 holotoxins were from Phoenix Laboratory at the Tufts Medical Center, Boston, MA, USA. They are purified by receptor analog affinity chromatography [38]. Ricin holotoxin was from Vector Laboratory, Burlingame, CA, USA. It was purified from castor bean (catalog number L-1090). Yeast monomeric ribosomes were purified as described previously [19]. The 10-mer SRL mimic oligo (5′-rCrGrCrGrArGrArGrCrG-3′) was purchased from Integrated DNA Technologies, Coralville, Iowa, IA, USA.

### 4.2. Toxin Structures

Stx (1R4Q), Stx2 (2GA4) and ricin (2AAI) holotoxins were downloaded from PDB. The A1 subunits of Stx and Stx2 or the A subunit of ricin were obtained from the holotoxins. The structures were visualized using PyMOL (The PyMOL Molecular Graphics System, Version 1.8 Schrödinger, LLC, Portland, OR, USA).

### 4.3. SDS-PAGE Analysis 

Stx1 or Stx2 holotoxin (7 µg) was treated with 50 ng TPCK-treated trypsin or trypsin plus 50 mM of TCEP at 25 °C for 2 h in a total volume of 10 µL in trypsin reaction buffer (50 mM Tris-HCl pH 8.0, 20 mM CaCl_2_). TPCK-treated trypsin was from Biolabs (Ipswich, MA, USA). Trypsin was reconstituted following the manufacturer’s instructions and used within a week. Ricin holotoxin (7 µg) was treated with 50 mM of TCEP at 25 °C for 2 h in 10 µL in its original buffer (10 mM of phosphate pH 7.8, 150 mM NaCl, and 0.08% sodium azide). 5 µL of 5X SDS-PAGE loading buffer was added to the treated and untreated Stx1, Stx2 or ricin. The samples were heated at 95 °C for 5 min and separated on 12% SDS-PAGE. The gel was stained with Coomassie blue.

### 4.4. Holotoxin-Ribosome Interaction

Holotoxins were immobilized on a CM5 chip of a Biacore T200. Ricin was immobilized on Fc2 at 1873 RU, Stx1 on Fc3 at 2541 RU and Stx2 on Fc4 at 1853 RU. Fc1 was activated and blocked as a control. Yeast ribosomes at 2.5, 5, 10, 20 and 40 nM were passed over the surface using single kinetic injection method. The running buffer contained 10 mM Hepes, pH 7.4, 150 mM NaCl, 5 mM MgCl_2_, 50 µM EDTA and 0.003% surfactant P20. The surface was regenerated by three times 1 min injection of 3 M NaCl. The interaction was measured at 25 °C.

### 4.5. Toxin Activation

The activation conditions were the same as used in the SDS-PAGE analysis described in Section 4.3. Stx1 and Stx2 were activated by treatment with trypsin and TCEP. At the end of the reaction 1 mM of PMSF was added to stop the reaction. The activated Stx1 or Stx2 was stored at 4 °C and used within two days. The activated ricin was also stored at 4 °C and used within two days. 

### 4.6. Ribosome Depurination

Ribosome depurination was measured using continuous enzyme coupled luminescent assay [34]. Ribosomes were used at 0.5 µM, Stx1 and Stx2 holotoxins were used at 0.2 nM and ricin was used at 1 nM. The reaction was started by adding the toxin, and luminescence intensity was recorded continuously for 15 min using a BioTek Synergy 4 Microplate reader (Winooski, VT, USA). Adenine standards were assayed at same time. The rates were determined from the linear region of the luminescence intensity. The adenine generated from ribosomes alone was subtracted.

### 4.7. SRL Depurination

Stem-loop depurination was performed using a synthetic 10-mer RNA mimic of the SRL. The discontinuous luminescence assay described by Sturm and Schramm was used [34]. In the reaction mixture, the SRL was used at 2 µM, Stxs at 5 nM or 1 µM and ricin at 40 nM or 1 µM. The reaction was incubated at 20 °C in 50 µL of 10 mM potassium citrate-KOH and 1 mM EDTA at pH 4.5 for both Stxs and ricin. The reaction was started by adding the toxins. Samples were taken at 1, 2 and 4 min and mixed immediately with 2X coupling buffer to stop the reaction. Adenine standards were assayed at the same time. Luminescence intensity was measured using a BioTek Synergy 4 Microplate reader.
