# Erratum: Differences in Ribosome Binding and Sarcin/Ricin Loop Depurination by Shiga and Ricin Holotoxins. *Toxins* **2017**, *9*, 133

**DOI:** 10.3390/toxins10030107

**Published:** 2018-03-01

**Authors:** Toxins Editorial Office

**Affiliations:** MDPI AG, St. Alban-Anlage 66, 4052 Basel, Switzerland; toxins@mdpi.com

We wish to make the following correction to the published paper [1]. The fifth sentence in Section 3 (Discussion) is incorrect. It should be as follows:

“Conserved arginines at the P-protein stalk binding site did not contribute to the differences in the affinity of the A1 subunits for the ribosome [32].”

The error was introduced during production, and is not the fault of the authors. We apologize for any inconvenience caused to the readers and authors by this change. The change does not affect the scientific results. The manuscript will be updated, and the original will remain available on the article webpage.
